# Pulmonary cryptococcosis with cavitation in an immunocompetent child: a case report and literature review

**DOI:** 10.3389/fped.2026.1821862

**Published:** 2026-06-09

**Authors:** Wen Zhang, Guohua Yao, Haoyi Wang, Juxia Xi, Cuian Ma, Botao Wei, Tongqiang Zhang

**Affiliations:** 1 Department of Infectious Diseases, Children's Hospital, Tianjin University/Tianjin Children's Hospital, Tianjin, China; 2 Tianjin Key Laboratory of Birth Defects for Prevention and Treatment, Children's Hospital, Tianjin University/Tianjin Children's Hospital, Tianjin, China; 3 Department of Respiratory Medicine, Children's Hospital, Tianjin University/Tianjin Children's Hospital, Tianjin, China; 4 Department of Respiratory Medicine, Shanxi Children's Hospital (Shanxi Women and Children Health Hospital), Taiyuan, China

**Keywords:** cavitation, child, cryptococcus, immunocompetent, pulmonary cryptococcosis

## Abstract

**Background:**

In recent years, the incidence of pulmonary cryptococcosis (PC) has risen among patients without human immunodeficiency virus (HIV) infection, including individuals with preserved immune function. The clinical manifestations of PC are largely non-specific, frequently presenting with fever, cough, chest tightness, or chest pain. In some cases, PC remains asymptomatic, which increases the likelihood of misdiagnosis or delayed recognition. The present study reports the diagnostic evaluation and therapeutic course of a child with PC and normal immune function, accompanied by a literature review, with the objective of enhancing clinical awareness and reducing disease-related mortality.

**Case Presentation:**

A 14-year-old girl was admitted to the hospital on April 28, 2025, with cough accompanied by intermittent fever. During the illness, chest tightness and chest pain were reported. Her past medical history was unremarkable. Physical examination demonstrated stable breathing, with scattered rales auscultated bilaterally. Chest computed tomography (CT) revealed extensive inflammatory consolidation with cavitary change in the right upper lobe. Cryptococcus neoformans capsular antigen (CrAg Lateral Flow Assay) was positive in both blood and bronchoalveolar lavage fluid (BALF), whereas cerebrospinal fluid (CSF) testing was negative. Targeted next-generation sequencing (tNGS) of BALF detected Cryptococcus neoformans (7,193 sequence reads), establishing the diagnosis of pulmonary cryptococcosis. Oral fluconazole at 6 mg/kg/day was administered as antifungal therapy. After 6 days of inpatient management, body temperature normalized and clinical symptoms markedly improved. The patient was discharged on day 9 in stable condition. Following discharge, she remained afebrile and free of cough, chest tightness, chest pain, or other complaints. Follow-up chest radiography at 16 weeks demonstrated near-complete resolution of the lesion, and repeat chest CT at 6 months showed absorption of the cavity with clear improvement compared with baseline; fluconazole was subsequently discontinued.

**Conclusion:**

PC may develop in children with preserved immune function. In pediatric patients presenting with pulmonary cavitation, non-specific respiratory manifestations, inadequate response to conventional anti-infective therapy, and suspected pulmonary tuberculosis or malignancy, cryptococcus-related etiologic investigations should be performed promptly. Treatment strategies should be individualized according to immune status and disease severity.

## 1 Introduction

Cryptococcus is an environmental saprophytic fungus for which pigeons serve as the natural reservoir, and soil contaminated with pigeon droppings constitutes a major source of transmission. Additional avian species and domestic animals may also harbor Cryptococcus spp. Pulmonary cryptococcosis (PC) represents a fungal infection of the lung resulting from inhalation of airborne Cryptococcus spores and occurs most frequently in immunocompromised individuals, particularly those infected with human immunodeficiency virus (HIV) (1). In 2022, Cryptococcus neoformans was designated by the World Health Organization (WHO) as the leading pathogenic fungal species (2). In recent years, the incidence of PC has increased substantially, with a growing proportion of cases reported in non-HIV-infected individuals and in patients with preserved immune function. Epidemiologic data in pediatric populations remain limited, especially in developing countries (3), which contributes to diagnostic difficulty. Previous studies have indicated that, among HIV-negative non-transplant hosts, the mean interval from symptom onset to diagnosis of cryptococcosis is 68 days (4). In the absence of timely recognition, PC may progress to disseminated infection and is associated with elevated mortality. Prompt diagnosis and appropriate antifungal therapy are associated with improved clinical outcomes. The present case involves an immunocompetent child with PC complicated by pulmonary cavitation and is intended to enhance clinical awareness and reduce disease-related mortality.

## 2 Case presentation

### 2.1 General information

A 14-year-old girl was admitted with a 12-day history of cough accompanied by intermittent fever. During the illness, chest tightness and chest pain were reported, without wheezing or dyspnea. The fever was low grade and intermittent, with a maximum temperature of 37.4 °C. Intravenous ceftizoxime had been administered at another hospital, with no significant clinical improvement. Past medical history was unremarkable. Immunizations had been administered according to schedule. Epidemiological investigation revealed that she was a boarding student living in an urban area, with no history of residence in rural or farm settings. She denied any contact with or raising of pigeons, other birds, or poultry. She had no hobbies associated with increased environmental exposure risk, such as sand digging, gardening, or outdoor field activities. In addition, she had no history of recent family travel or outings. No clear epidemiological clues suggestive of Cryptococcus infection were identified.

### 2.2 Clinical manifestations

At admission, physical examination revealed a thin body habitus, appropriate growth and development, preserved mental status, and stable respiration. A positive BCG scar was noted. Pharyngeal congestion was present, and scattered rales were auscultated in both lungs. Cardiovascular, abdominal, and neurologic examinations showed no significant abnormalities.

### 2.3 Auxiliary examinations

Chest CT performed on April 24, 2025, demonstrated extensive inflammatory consolidation with a small cavitary component in the right upper lobe (Figure 1). Following admission, cough persisted and was accompanied by chest tightness, dyspnea, and recurrent fever, with a peak temperature of 38 °C. A comprehensive diagnostic evaluation was undertaken, and flexible bronchoscopy was completed on the first hospital day. Electrocardiography, echocardiography, and pulmonary function testing yielded normal results. Chest radiography revealed patchy high-density opacities in the upper zone of the right lung. Bronchoscopy showed inflammatory changes involving the bronchus of the right upper lobe (Figure 2). Ultrasonography demonstrated no pleural effusion and no significant abnormalities of the liver, spleen, or kidneys. Complete blood count showed hemoglobin 129 g/L, white blood cell 15.19 × 10⁹/L, neutrophil percentage 76%, lymphocyte percentage 17%, monocyte percentage 6%, eosinophil percentage 1%, and platelet 311 × 10⁹/L. C-reactive protein was 39.14 mg/L, and erythrocyte sedimentation rate was 47 mm/h. Serum biochemistry, including electrolytes, glucose, hepatic and renal function, and muscle enzymes, remained largely within normal limits. Lymphocyte subsets, immunoglobulin concentrations, and complement levels were normal. Serologic testing for hepatitis, HIV, and tuberculosis infection T-cell assay was negative, and blood cultures showed no growth. Cryptococcus capsular antigen (CrAg) was positive in both blood and bronchoalveolar lavage fluid (BALF). CrAg testing was performed using the Immy lateral flow assay (LFA) kit, which only provides qualitative (positive/negative) results and cannot be used for quantitative determination of CrAg titers, thus having certain limitations. Targeted next-generation sequencing (tNGS) of BALF identified Cryptococcus neoformans. India ink staining of BALF for Cryptococcus neoformans was negative. Liquid-based cytology of BALF demonstrated neutrophils 55%, eosinophils 0%, lymphocytes 1%, macrophages 42%, and epithelial cells 2%. To exclude cryptococcal meningitis, lumbar puncture with cerebrospinal fluid (CSF) analysis was performed on the second hospital day. CSF routine parameters and biochemistry were normal, CSF CrAg testing was negative, Gram stain smear and India ink staining were negative, and CSF tNGS and culture yielded negative results. Cranial MRI showed no significant abnormalities.

**Figure 1:**
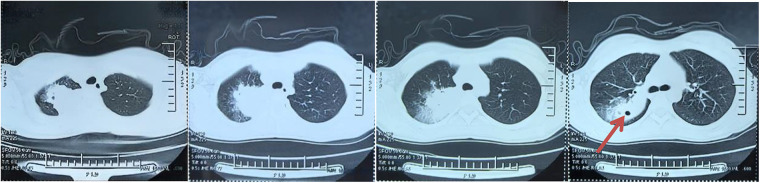
Chest CT plain scan obtained on April 24, 2025, demonstrating extensive inflammatory consolidation with a small cavitary lesion in the right upper lobe.

**Figure 2:**
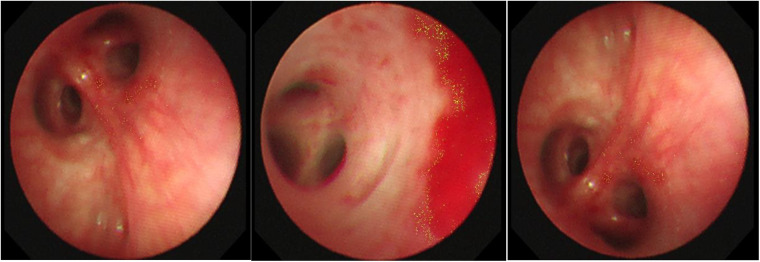
Bronchoscopic evaluation of the right upper lobe (RUL), with inflammatory changes observed in the anterior and apicoposterior segments. Right Upper Lobe (RUL), Anterior segment (RUL), Apicoposterior segment (RUL).

### 2.4 Diagnosis

In light of the patient's clinical presentation, supportive ancillary findings—including positive CrAg in blood and BALF, identification of Cryptococcus neoformans by BALF tNGS, and characteristic chest CT features—and exclusion of alternative diagnoses such as tuberculosis and malignancy, pulmonary cryptococcosis was established on the second hospital day.No evidence of extrapulmonary dissemination was identified. Given her previously healthy status, absence of clinical manifestations suggestive of immunodeficiency, lack of extrapulmonary dissemination, and normal initial immunological workup, further advanced immunological tests, including lymphocyte proliferation assays, were not clinically indicated at that time.

### 2.5 Treatment and prognosis

Following diagnostic confirmation, oral fluconazole was initiated at 6 mg/kg/day for antifungal therapy. After 6 days of treatment, body temperature normalized, and respiratory symptoms, including cough, chest tightness, and dyspnea, improved. Repeat chest radiography demonstrated partial resolution of pulmonary infiltrates compared with prior imaging. The patient was discharged on hospital day 9 in stable condition. During outpatient follow-up, she remained afebrile and free of cough, chest tightness, or other discomfort. Chest CT at 6 months confirmed resolution of the cavitary lesion with radiologic improvement relative to baseline (Figure 3). Fluconazole therapy was subsequently discontinued. The treatment course is summarized in Figure 4.

**Figure 3:**
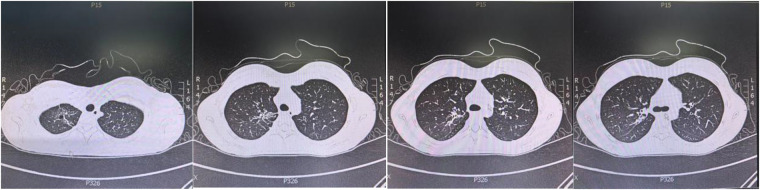
Chest CT plain scan performed on November 6, 2025, indicating resolution and radiologic improvement of the cavitary lesion compared with prior imaging.

**Figure 4:**
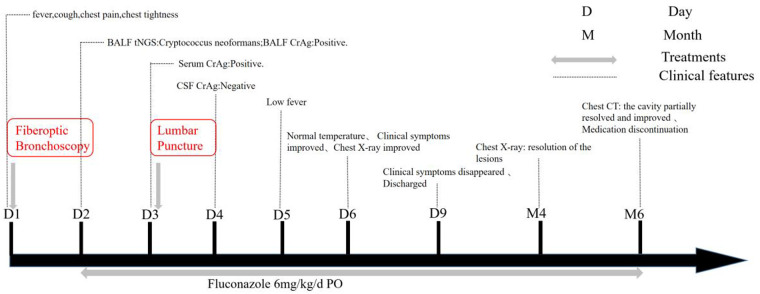
Therapeutic course.

### 2.6 Imaging findings

## 3 Discussion

Prior reports have indicated that cryptococcosis occurs most frequently in immunocompromised pediatric patients. In recent years, however, an increasing number of cases have been documented among children without evident underlying immunodeficiency (5). A meta-analysis by Zhang XB et al. of 116 non-AIDS pediatric cryptococcosis cases drawn from 45 domestic and international studies reported immunodeficiency in only 12.9% of patients (3.4% in domestic cases and 9.5% in foreign cases). Organ involvement most commonly affected the lungs (77.6%), central nervous system (53.4%), and lymph nodes (51.7%), followed by the liver (48.3%), spleen (37.9%), and skin (21.6%) (6).Thirteen case reports of cryptococcosis in immunocompetent children published within the past 5 years were reviewed (6–13) (Table 1). The cohort included 10 males and 3 females, with a mean age of 9.31 years. Male predominance was observed, consistent with findings reported by Chen LA et al. (14). This sex disparity may be partly attributable to hormonal modulation by estrogen. Experimental data have suggested that Cryptococcus exhibits slower growth in females, as capsular shedding occurs more readily in a testosterone milieu than under exposure to 17-β estradiol (15). Among the 13 cases, 5 represented isolated pulmonary cryptococcosis, whereas 8 were disseminated infections. Affected sites included the lungs (13 cases), lymph nodes (5 cases), liver (2 cases), spleen (2 cases), skin (1 case), bone marrow (1 case), and pericardium (1 case). Pulmonary involvement predominated, and central nervous system dissemination was not identified, consistent with the present case. In a study of 234 non-HIV-infected PC patients, Chen M et al. reported a lower risk of neurologic involvement in immunocompetent individuals compared with immunocompromised hosts (16). All 13 children had been previously healthy, with normal humoral and cellular immune profiles and no documented exposure to pigeon droppings. Li Xiaomeng et al. summarized 18 reports of pulmonary cryptococcosis in immunocompetent patients, similarly noting preserved immune status and absence of pigeon-dropping exposure, indicating that neither immunodeficiency nor environmental contact should be regarded as the primary diagnostic clue for this disease (17).

**Table 1:** Clinical data of 13 cases of cryptococcosis in immunocompetent children reported in recent 5 years.

Author	Year	Gender	Age	Main symptoms	Invaded organ	Diagnostic methods	Risk factors	Treatment	Outcome
Vijayasekharan et al. (7)	2021	F	11y	Fever, abdominal pain, respiratory distress, jaundice	Lung, LD, Liver, Spleen	CT: bilateral areas of air-space opacifications with surrounding ground-glass opacities; Histopathologic examination of LD biopsy; CrAg: Positive	No	AMB+5-FC + FZ (12month)	Improved
Zhang et al. (6)	2025	M	5y	Fever, cough, rash	Lung, BM, LD	CT: bilateral lung infection with multiple calcifications in the right upper lobe and hilar lymph nodes; CrAg: Positive (1:5); BM Culture: Cryptococcus neoformans; Metagenomic Pathogen Detection (LP Necrotic Pus): Cryptococcus neoformans	No	AMB + FZ + VC (10month)	Improved
Dai et al. (8)	2024	M	13y	Cough, chest pain	Lung	CT: a lesion and cavities in the right lower lobe of the lung; Serum CrAg: Positive; BALF NGS: Cryptococcus species	No	FZ (6month)	Improved
Li et al. (9)	2025	M	15y13y6y	Chest pain	Lung	Chest CT: multiple nodules, high-density shadows and nodules; BALF Culture: Cryptococcus neoformans; Lung biopsy pathological tissue culture: Cryptococcus neoformans; Serum CrAg: Positive	No	FZ (6month)	Cured
Hu and Wang (10)	2022	M	15y	Fever, cough, abdominal pain, rash, Subcutaneous nodules	Lung, LD, Skin	Chest CT: scattered miliary nodular opacities in both lungs; LD and skin biopsy pathology: Cryptococcus	No	FZ (8month)	Improved
Nakatudde et al. (3)	2021	M	8y	Cough, fever, night sweats, weight loss, chest pain, difficulty in breathing	Lung, Pericardium	Chest CT: an extensive hetero-complex mass in the right hemi-thorax with pericardial invasion; Lung biopsy pathological: Cryptococcus; Serum CrAg: Positive	No	AMB + FZ (lost to follow-up)	Improved
Zhang et al. (11)	2021	2M,1F	3y 7y 10y	Cough, fever, cervical lymphadenopathy	Lung, LD, Spleen	Chest CT: 3 cases all showed significant multiple mediastinal lymphadenopathy, 1 case was accompanied by scattered pulmonary nodules; 2 cases Serum CrAg: Positive(1:16.1:64); 2 cases LD biopsy pathology: Cryptococcus neoformans	one case of pigeons kept by a neighbor	1 case AMB + FZ, (5month) 2 cases FZ (6/9month)	Cured
Qiu et al. (12)	2020	M	13y	Cough, back pain	Lung	Chest CT: a mass-like lesion in the left upper lobe of the lung; Quantitative determination of serum Cryptococcus neoformans capsular polysaccharide:777.6 μg/L.Culture of BALF: Cryptococcus species	No	FZ (6month)	Improved
Zhang et al. (13)	2021	F	2y	Fever	Lung, Blood, LD, Liver	Chest CT: patchy and nodular hyperdense opacities are visible, with widening of the superior mediastinum and obviously enlarged lymph nodes seen anterior to the trachea; Culture of BALF: Cryptococcus species; LD and lung biopsy pathology: Cryptococcus neoformans	No	VC + LAMB+5-FC + FZ (12month)	Cured

Abbreviations: LD, lymph node; BM, Bone Marrow; LP, lung puncture; ITP, idiopathic thrombocytopenic purpura; LAMB, Liposomal amphotericin B; AMB, amphotericin-B; 5-FC, 5-flucytosine; FC, flucytosine; FZ, fluconazole; VC, voriconazole.

The clinical spectrum of PC is broad, ranging from asymptomatic infection to acute respiratory distress syndrome, with disease severity largely determined by host immune status. In immunocompetent patients, manifestations are generally mild and most often include cough, fever, and chest pain, with lesions typically confined to the lungs. By contrast, immunocompromised individuals or those with significant comorbidities carry a substantially greater risk of disseminated cryptococcosis (18). Radiologic features of pulmonary cryptococcosis remain non-specific. As observed in the present case, cavitary pulmonary lesions may occur, with a reported incidence of approximately 10.96–11.2% (14, 16). A separate report described a 16-year-old Chinese girl receiving long-term glucocorticoid therapy who developed bilateral diffuse cavitary nodules (19). Additional imaging patterns include solitary or multiple nodules or masses, parenchymal infiltrates, interstitial changes, diffuse mixed lesions, and pleural effusion (20). Because these findings may closely resemble lung cancer, pulmonary tuberculosis, or sarcoidosis, missed or incorrect diagnoses are common. Nakatudde I et al. documented a pediatric PC case initially misdiagnosed as pulmonary tuberculosis (3). In the current patient, admission was prompted by cough, chest tightness, and low-grade fever, with chest CT demonstrating consolidation and cavitation and limited response to conventional anti-infective therapy. In immunocompetent children presenting with pulmonary cavitation and non-specific respiratory symptoms, PC should be incorporated into the differential diagnosis to prevent delayed recognition and unnecessary investigations.

The diagnosis of PC is established through integration of clinical manifestations, radiologic features, pathologic assessment, and etiologic evidence, with definitive confirmation primarily dependent on microbiologic and histopathologic findings. In the present case, the patient initially received empirical antimicrobial therapy covering bacterial pathogens and Mycoplasma pneumoniae according to clinical presentations. However, neither clinical symptoms nor radiological findings improved, and persistent cavitary lesions on chest CT raised suspicion of uncommon pathogens such as fungi. Conventional smears and cultures yield low detection rates, and positivity from BALF specimens has been reported in fewer than 10% of cases, especially in patients with isolated pulmonary cryptococcosis (21). Routine fungal culture of bronchoalveolar lavage fluid was performed in this case, and the result was negative, which is consistent with previous reports. Consequently, percutaneous lung aspiration or biopsy is frequently required to obtain tissue for pathologic confirmation. Such procedures, however, are limited by prolonged turnaround times and the inherent invasiveness of tissue acquisition, which may complicate routine clinical management.

In recent years, detection of CrAg has gained increasing recognition as a rapid and sensitive diagnostic approach for PC and is recommended in current guidelines (22). In the present study, CrAg detection was performed using the Immy cryptococcal antigen lateral flow assay (LFA) kit. This method shows favorable diagnostic sensitivity and specificity in non-HIV-infected patients with isolated pulmonary cryptococcosis. In contrast, the latex agglutination assay has suboptimal sensitivity in this population, and a negative serum CrAg result by this method does not exclude infection, which may lead to delayed clinical diagnosis (23). For the CrAg lateral flow assay (LFA), one study reported an overall sensitivity of 97.6% and specificity of 98.1% (24), respectively, for serologic detection of Cryptococcus infection. CrAg titers correlate positively with the quantity of antigen released from cryptococcal lesions into the circulation. In a cohort of 107 patients with localized pulmonary cryptococcosis (25), multivariate analysis demonstrated a positive association between CrAg titers and both the number and size of pulmonary lesions. Following effective antifungal therapy, progressive reduction in lesion size was accompanied by a sustained decline in CrAg titers. Collectively, this evidence supports the use of cryptococcal antigen not only as a diagnostic marker for pulmonary cryptococcosis but also as an adjunct for clinical assessment of disease burden and therapeutic response in localized cases. Nevertheless, low-level titers may persist for an extended period after successful treatment. In the present case, serum CrAg remained positive 6 months after clinical and radiologic improvement. Persistent antigenemia is attributed to continued release of capsular polysaccharide from nonviable organisms and relatively slow host clearance. Accordingly, CrAg titers may serve as an indicator of antifungal response but should not be regarded as definitive evidence of cure or as the sole criterion for treatment discontinuation.

With the expanding application of tNGS, this modality has been increasingly incorporated into clinical practice. By performing high-throughput sequencing of specific genomic regions using designed probes or primers, tNGS enables precise pathogen identification, rapid quantification, and acquisition of detailed genetic information, thereby enhancing the detection rate of Cryptococcus and offering substantial clinical utility (26). In contrast to metagenomic next-generation sequencing (mNGS), tNGS focuses on a predefined panel of common respiratory pathogens, including bacteria, mycoplasma, fungi, and mycobacteria. This panel is sufficient for most routine pediatric respiratory infections and allows more rapid and focused interpretation. In addition, tNGS is less costly than mNGS, thereby reducing the economic burden for the patient and family. For these reasons, tNGS was chosen as the first-line molecular diagnostic approach in this case.In earlier reports, tNGS was infrequently applied for definitive confirmation of PC. A 30-year clinical analysis of pulmonary cryptococcosis in non-AIDS patients at a tertiary hospital in Beijing noted that bronchoscopy had not been performed in any of the misdiagnosed cases. This pattern was presumed to reflect reliance on CT findings and clinical judgment when guiding therapy, with bronchoscopy not yet routinely incorporated into diagnostic pathways for pulmonary cryptococcosis. In the present case, diagnosis was established through detection of Cryptococcus capsular antigen and BALF tNGS, with additional cryptococcal burden data obtained (27). This approach contributed to timely recognition and provided supportive evidence for improving future diagnostic strategies and clinical outcomes in PC. Nonetheless, false-negative tNGS results remain possible. Yu et al (28). reported a case of pulmonary cryptococcosis with negative BALF sequencing despite positive CrAg testing. Such discordance may be explained by the thick lipid-rich outer wall of Cryptococcus neoformans, which complicates nucleic acid extraction and limits complete genomic recovery. Furthermore, pathogen concentrations may fall below the sequencing detection threshold, or sequencing depth may be inadequate for reliable identification of the target organism. Therefore, negative tNGS results should not be interpreted as definitive exclusion of Cryptococcus infection, and combined antigen testing is recommended to support diagnostic accuracy and enable prompt initiation of antifungal therapy. In pediatric patients with positive serum CrAg, additional CSF CrAg testing is advised to exclude disseminated disease, including cryptococcal meningitis.

In immunocompetent adults, PC may occasionally resolve without intervention; however, pediatric immune function remains relatively immature, which increases the potential for progression and dissemination. Accordingly, antifungal therapy is generally advised for children with PC. The primary objective of treatment is eradication of infection and prevention of systemic spread, with regimen selection guided by immune status and disease severity. Fluconazole is characterized by high bioavailability and a favorable safety profile, and is therefore recommended as first-line therapy for PC in immunocompetent pediatric patients (29). The 2024 Global Guidelines for Cryptococcosis propose severity-based management of pulmonary cryptococcosis, stratified by mild versus severe disease and by the presence or absence of pulmonary cryptococcoma (30). Based on imaging features, the present patient met criteria for severe disease, for which induction, consolidation, and maintenance therapy would typically be advised. Nevertheless, preserved immune function, mild clinical manifestations, and absence of dissemination supported selection of fluconazole monotherapy in consideration of potential drug-related toxicity. Clinical symptoms and radiologic abnormalities improved within 5 days of treatment initiation. Similarly, Li et al. (9) administered fluconazole alone to three immunocompetent children with PC classified as severe on imaging, all of whom achieved favorable outcomes. Collectively, these observations suggest that treatment thresholds may be individualized in immunocompetent pediatric patients, with therapeutic decisions optimized according to clinical presentation and response.

A total of 13 pediatric cryptococcosis cases with normal immune function were summarized in Table 1. These cases shared consistent clinical features: insidious onset with mild symptoms (mainly fever, chronic cough, chest pain), normal immune indicators including immunoglobulins, lymphocyte subsets and CD4+/CD8 + counts, no evidence of primary immunodeficiency or HIV infection. The main radiological patterns were pulmonary nodules, masses, consolidation, cavitary lesions and mediastinal lymphadenopathy. Most cases were confirmed by CrAg, fungal culture, BALF tNGS/mNGS or tissue biopsy. Most patients had no clear exposure history to pigeons, poultry, soil or epidemic areas, suggesting that environmental exposure is not a necessary diagnostic clue. Cryptococcus neoformans was the main pathogen, rather than Cryptococcus gattii, which is classically considered more likely to infect immunocompetent hosts. This indicates that C. neoformans has become an important pathogen of pulmonary cryptococcosis in immunocompetent children. All cases were treated with antifungal therapy (mostly fluconazole monotherapy) for 6–12 months and achieved favorable outcomes.

Therefore, with the rising incidence of PC among immunocompetent children, the assumption that preserved immunity confers minimal risk warrants reconsideration. In children presenting with non-specific manifestations, limited response to standard anti-infective therapy, and pulmonary cavitation raising suspicion for tuberculosis or malignancy, early implementation of Cryptococcus capsular antigen testing and BALF high-throughput sequencing may reduce diagnostic error, lessen patient burden and healthcare expenditures, and inform subsequent investigative efforts. In this population, therapeutic indications may be judiciously individualized, and an appropriate regimen should be initiated promptly according to clinical characteristics to optimize outcomes. Establishment of a dedicated pediatric cryptococcosis registry network is also proposed to enable systematic surveillance of long-term prognosis.

## Data Availability

The original contributions presented in the study are included in the article/Supplementary Material, further inquiries can be directed to the corresponding author/s.
