# Correction: Pulmonary cryptococcosis with cavitation in an immunocompetent child: a case report and literature review

**DOI:** 10.3389/fped.2026.1911445

**Published:** 2026-07-02

**Authors:** Wen Zhang, Guohua Yao, Haoyi Wang, Juxia Xi, Cuian Ma, Botao Wei, Tongqiang Zhang

**Affiliations:** 1 Department of Infectious Diseases, Children’s Hospital, Tianjin University/Tianjin Children’s Hospital, Tianjin, China; 2 Tianjin Key Laboratory of Birth Defects for Prevention and Treatment, Children’s Hospital, Tianjin University/Tianjin Children’s Hospital, Tianjin, China; 3 Department of Respiratory Medicine, Children’s Hospital, Tianjin University/Tianjin Children’s Hospital, Tianjin, China; 4 Department of Respiratory Medicine, Shanxi Children’s Hospital (Shanxi Women and Children Health Hospital), Taiyuan, China

**Keywords:** cavitation, child, cryptococcus, immunocompetent, pulmonary cryptococcosis

In the published article, the statement for equal contributions and joint first authorship of the first three authors was missing.

The following statement has been added for authors Wen Zhang, Guohua Yao and Haoyi Wang.

“These authors have contributed equally to this work and share first authorship”

The original article has been updated.

